# Influence of Smoking on the Location of Acute Myocardial Infarctions

**DOI:** 10.5402/2011/174358

**Published:** 2011-04-17

**Authors:** Rahel Alemu, Eileen E. Fuller, John F. Harper, Mark Feldman

**Affiliations:** Department of Internal Medicine, Texas Health Presbyterian Hospital of Dallas, 8200 Walnut Hill Lane, Dallas, TX 75231, USA

## Abstract

*Objective*. To determine whether there is an association between smoking and the location of acute myocardial infarctions. *Methods*. Using a cohort from our hospital and published cohorts from Ireland, Uruguay, and Israel, we calculated odds of having an inferior wall as opposed to an anterior wall acute myocardial infarction among smokers and nonsmokers. *Results*. In our cohort, there was a higher proportion of smokers than nonsmokers in patients with inferior acute myocardial infarctions than in patients with anterior infarctions. This difference was also present in each of the other cohorts. Odds ratios for an inferior versus an anterior acute myocardial infarction among smokers ranged from 1.15 to 2.00 (median odds ratio, 1.32). When the cohorts were combined (*n* = 3, 160), the pooled odds ratio for an inferior as opposed to an anterior acute myocardial infarction among smokers was 1.38 (95% confidence interval, 1.20 to 1.58) (*P* < .002). *Conclusions*. Cigarette smoking increases the risk of inferior wall acute myocardial infarction more than the risk of anterior wall infarction. Smoking thus appears to adversely affect the right coronary arterial circulation to a greater extent than the left coronary arterial circulation by a mechanism not yet understood.

## 1. Introduction

Cigarette smoking is a major, independent risk factor for coronary heart disease (CHD) and acute myocardial infarction (AMI) [1, 2]. The mechanism for the adverse effect of cigarette smoking on the coronary arterial circulation is complex and multifactorial. 

Smoking increases both heart rate and blood pressure (and therefore the rate-pressure product), thereby augmenting myocardial oxygen demand. Simultaneously, smoking reduces the dimension of the coronary arteries and coronary blood flow [3]. Whether smoking-induced coronary vasoconstriction and reduced coronary blood flow affect the right and left coronary arterial systems equally and whether smoking constricts normal and diseased arteries to a similar degree is uncertain. 

Inferior wall AMI is usually a consequence of disease in the right coronary arterial system, whereas anterior wall AMI is usually a consequence of disease in the left coronary arterial system. The purpose of the present study was to examine whether smoking influences the location of AMI (inferior wall versus anterior wall). To accomplish this purpose, we examined a recent cohort of patients with AMI from our own institution, all of whom had undergone coronary angiography at the time of their AMI [4]. We also examined data from published cohorts from Ireland, Uruguay, and Israel [5–8]. We employed identical methodologies to extract data from these reports and to calculate the odds of having an inferior as opposed to an anterior AMI among smokers and nonsmokers.

## 2. Methods

Details of our cohort of AMI patients have been described elsewhere [4]. Most of the patients (174, or 98%) had presented with inferior or anterior wall STEMI (98%); the other 4 (2%) had presented with AMI associated with left bundle branch block. Every patient in this cohort underwent coronary angiography, almost always emergently, and the infarct-related culprit artery was identified (Table 1). As anticipated, the right coronary artery was the culprit in over 80% of the patients with inferior AMI, while the left anterior descending artery was the culprit in over 95% of the patients with anterior AMI.

We also searched the print and online literature for other AMI cohorts that, like ours, were classified as inferior or anterior AMI patients and also as smokers or nonsmokers. Four additional cohorts, listed in Table 2, fulfilled these criteria [5–8]. 

We uniformly extracted data from the text or tables of each report. We expressed data as percentages of smokers in the inferior and the anterior AMI groups. For each study, and for the pooled cohorts, we calculated the odds ratio (and its 95% confidence interval) of having an inferior AMI relative to an anterior AMI among smokers. We also calculated the *z*-ratio and the corresponding *P* value using pooled data.

## 3. Results

As shown in Table 3, the prevalence of smoking differed considerably among the five cohorts analyzed. The prevalence of smoking ranged from 41.9% to 84.7% among patients with inferior AMI and from 36.4% to 74.4% among patients with anterior AMI. In each of the cohorts examined, however, smokers were overrepresented in the inferior AMI groups compared to the anterior AMI groups. 

Odds ratios for an inferior relative to anterior AMI among smokers in the five cohorts ranged from 1.15 to 2.00 (median, 1.32). The pooled odds ratio for the combined cohorts was 1.38 (95% confidence interval, 1.20–1.58). This difference was statistically significant (*z*-ratio, 4.491; 2*P* < .002). The pooled data reflected an 8% higher incidence of smoking in inferior versus anterior AMI patients (95% confidence interval for the difference, 4.5–11.4%).

## 4. Discussion

The purpose of our study was to accept or reject the null hypothesis that smoking would be equally associated with inferior and anterior wall AMI locations. In each of the cohorts that we analyzed, spanning three decades, there were higher percentages of smokers among patients with inferior than anterior AMI. Conversely, nonsmokers were overrepresented among patients with anterior AMI. Similar trends have been observed in clinical trials involving AMI patients [9–11]. The consistent difference in smoking prevalence by infarct location is intriguing and supports a hypothesis that smoking may adversely affect the right coronary arterial circulation to a greater extent than the left circulation. However, the mechanism(s) responsible for this apparent selective effect of smoking in CHD remains speculative.

Cigarette smoking increases the risk for AMI by multiple and complex mechanisms. With respect to atherogenesis, smoking raises serum LDL-cholesterol and triglyceride concentrations and lowers serum HDL-cholesterol levels. Moreover, cigarette smoke promotes free radical damage to LDL, leading to accumulation of oxidized LDL-cholesterol within the arterial wall. Smoking appears to contribute to the vascular inflammation characteristic of atherosclerosis, as reflected by higher serum C-reactive protein levels in smokers than in nonsmokers [1]. Whether these atherogenic processes are more prominent in the right than in the left coronary arterial circulation is uncertain. Some of the adverse effects of smoking on atherogenesis may not be rapidly reversible following smoking cessation, as progression of atherosclerosis appears to occur at similar rates in current and former smokers [12]. 

Smoking, mainly through its nicotine content, activates the sympathetic nervous system (SNS), increasing both heart rate and systolic blood pressure. This increase in the rate-pressure product results in increased myocardial oxygen demands. Increased SNS activity due to nicotine exposure also leads to coronary arterial vasoconstriction [3], decreasing myocardial blood flow at a time when oxygen demand is increasing. In addition to increasing myocardial oxygen demand and reducing coronary blood flow, cigarette smoking increases carboxyhemoglobin levels in the blood, with the potential to further reduce myocardial oxygen delivery from oxyhemoglobin. Whether coronary arterial vasoconstriction induced by cigarette smoking is more pronounced in the right than the left coronary circulation is unknown. Nasal cocaine administration leads to similar degrees of vasoconstriction in the left and right coronary arterial systems [13]. Unfortunately, a similar comparison has not been performed before and after cigarette smoking. 

Smoking also impairs endothelial function, impairing release of tissue plasminogen activator (tPA) and prostacyclin (PGI_2_), for example, which could result in local hypercoagulability. Cigarette smoking may further contribute to hypercoagulability by increasing tissue factor, factor VII, fibrinogen, and hemoglobin levels. Smoking also enhances platelet activity and interactions between platelets and the endothelial lining of blood vessels. Whether any of these adverse endothelial and/or prothrombotic effects would occur to a greater extent in the right than the left coronary arterial system is a matter of speculation. Smoking also reduces endothelial release of nitric oxide (NO), which leads to reduced coronary flow reserve.

In summary, we found that cigarette smoking is associated more strongly with inferior than anterior AMI. This finding suggests that the adverse effects of tobacco on coronary atherogenesis and/or endothelial dysfunction may be more pronounced in the right than the left coronary arterial circulation. Despite the statistically significant association with smoking and the location of an AMI, the difference in smoking prevalence was modest (8%; 95% CI, 4.5–11.4%). Furthermore, there was still nearly a 50% prevalence of smoking among pooled patients with anterior AMI, which tend to be larger, and more lethal, than inferior AMI. This latter observation emphasizes the importance of smoking in the pathogenesis of both inferior and anterior AMI and the critical role of smoking avoidance and smoking cessation in primary and secondary prevention of AMI [14]. Since passive smoking exposure increases the risk of CHD and AMI in nonsmokers, smoking cessation can also substantially reduce the risk of AMI in nonsmokers [14].

## Figures and Tables

**Table 1:** Dallas AMI cohort [4]: culprit artery in relation to the location of the AMI.

	Inferior AMI (*n* = 107)	Anterior AMI (*n* = 71)
RCA	88 (82.2%)*	0
LCX	13 (12.1%)	2 (2.8%)
LAD	4 (3.7%)	69 (97.1%)^†^
LCX/LAD	1 (0.9%)	0
Ramus branch	1 (0.9%)	0

AMI: acute myocardial infarction; LAD: left anterior descending artery or its branches; LCX: left circumflex artery or its branches; RCA: right coronary artery or its branches.

*a single anomalous RCA supplied the entire left ventricle in one inferior AMI patient.

^†^Distal left main/LAD lesion was the culprit artery in one patient, LAD/ramus in another.

**Table 2:** Definitions of smokers and nonsmokers used in the AMI cohorts.

Reference	Country	AMI patients	Definition of smokers	Definition of nonsmokers
[4]	USA	178	Current and former smokers	Lifelong nonsmokers
[5]	Ireland	697	Current and former smokers	Lifelong nonsmokers
[6]	Uruguay	788	Current and former smokers	Lifelong nonsmokers
[7]	Israel	637	Current smokers and recent quitters*	Lifelong nonsmokers and former smokers^†^
[8]	Israel	666	Not stated	Not stated

*Patients who had quit smoking less than 1 month prior to their AMI.

^†^Patients who had quit smoking more than 1 month prior to their AMI.

**Table 3:** Smokers (%) among inferior and anterior AMI patients in the five cohorts.

Reference	Inferior AMI (%)	Anterior AMI (%)	Odds ratio (95% CI)
[4]	67.3	50.7	2.00 (1.08–3.70)
[5]	84.7	74.4	1.90 (1.30–2.78)
[6]	43.2	36.5	1.32 (1.03–1.73)
[7]	41.9	36.4	1.26 (0.92–1.74)
[8]	47.5	44.1	1.15 (0.85–1.57)
*Pooled*	*54.85*	*46.86*	*1.38 (1.20–1.58)*

AMI: acute myocardial infarction; CI: confidence interval.
